# Tetra­aqua­bis{2-[4-(3-pyrid­yl)pyrimidin-2-ylsulfan­yl]acetato}manganese(II) dihydrate

**DOI:** 10.1107/S1600536809033078

**Published:** 2009-08-22

**Authors:** Hai-Bin Zhu, Gang Xu, Yan-Yan Sun

**Affiliations:** a School of Chemistry and Chemical Engineering, Southeast University, Nanjing 211189, People’s Republic of China

## Abstract

In the title compound, [Mn(C_11_H_8_N_3_O_2_S)_2_(H_2_O)_4_]·2H_2_O, the Mn^II^ ion lies on an inversion centre and is coordinated by four water mol­ecules in equatorial positions and two N atoms from two 2-[4-(3-pyrid­yl)pyrimidin-2-ylsulfan­yl]acetate ligands in the axial positions. The water mol­ecules, including the uncoordinated water mol­ecules, and the acetate O atoms are involved in O—H⋯O and O—H⋯N hydrogen-bonding inter­actions, which link the components into layers parallel to the *a* (*b* + *c*) plane.

## Related literature

For hydro­(solvo)thermal reactions between (heterocyclic­thio)acetic acid and metal ions, see: Zhu *et al.* (2009 ▶); Hao *et al.* (2008 ▶); He *et al.* (2007 ▶). For a Cu(II) coordination compound with 4-(pyridin-4-yl)pyrimidine-2-sulfonate, see Li *et al.* (2009 ▶).
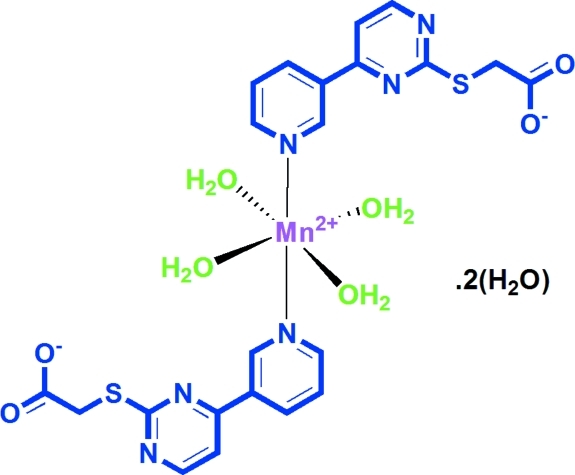


## Experimental

### 

#### Crystal data

[Mn(C_11_H_8_N_3_O_2_S)_2_(H_2_O)_4_]·2H_2_O
*M* *_r_* = 655.58
Triclinic, 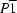

*a* = 8.459 (3) Å
*b* = 9.240 (3) Å
*c* = 9.360 (4) Å
α = 87.396 (6)°
β = 75.862 (5)°
γ = 79.872 (5)°
*V* = 698.4 (4) Å^3^
*Z* = 1
Mo *K*α radiation
μ = 0.69 mm^−1^
*T* = 298 K
0.14 × 0.12 × 0.10 mm

#### Data collection

Bruker APEXII CCD area-detector diffractometer
Absorption correction: multi-scan (*SADABS*; Bruker, 2001 ▶) *T*_min_ = 0.884, *T*_max_ = 0.920
4518 measured reflections
3181 independent reflections
2443 reflections with *I* > 2σ(*I*)
*R*_int_ = 0.031

#### Refinement

*R*[*F* ^2^ > 2σ(*F* ^2^)] = 0.037
*wR*(*F* ^2^) = 0.094
*S* = 0.98
3181 reflections
187 parameters
H-atom parameters constrained
Δρ_max_ = 0.49 e Å^−3^
Δρ_min_ = −0.55 e Å^−3^

### 

Data collection: *APEX2* (Bruker, 2007 ▶); cell refinement: *SAINT-Plus* (Bruker, 2007 ▶); data reduction: *SAINT-Plus*; program(s) used to solve structure: *SHELXS97* (Sheldrick, 2008 ▶); program(s) used to refine structure: *SHELXL97* (Sheldrick, 2008 ▶); molecular graphics: *SHELXTL* (Sheldrick, 2008 ▶); software used to prepare material for publication: *SHELXTL*.

## Supplementary Material

Crystal structure: contains datablocks I, global. DOI: 10.1107/S1600536809033078/cv2600sup1.cif

Structure factors: contains datablocks I. DOI: 10.1107/S1600536809033078/cv2600Isup2.hkl

Additional supplementary materials:  crystallographic information; 3D view; checkCIF report

## Figures and Tables

**Table 1:** Hydrogen-bond geometry (Å, °)

*D*—H⋯*A*	*D*—H	H⋯*A*	*D*⋯*A*	*D*—H⋯*A*
O2—H1⋯O3^i^	0.85	1.82	2.655 (2)	168
O1—H2⋯O4	0.85	1.88	2.709 (2)	165
O2—H3⋯O4	0.85	1.97	2.743 (2)	150
O1—H4⋯O5^ii^	0.85	1.81	2.642 (3)	167
O5—H5⋯N1^iii^	0.85	2.09	2.888 (3)	155
O5—H6⋯O3	0.85	2.01	2.775 (3)	149

Symmetry codes: (i) 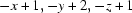; (ii) 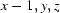; (iii) 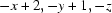.

**Fig. 1.**
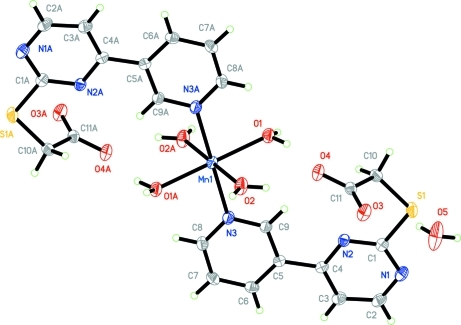
The coordination environment around Mn(II) in the title complex with the atom-labeling scheme [symmetry code: (A) -x, 2-y, 1-z]. Displacement ellipsoids for non-hydrogen atoms are drawn at the 30% probability level.

## supplementary crystallographic information

### Comment

Hydro(solvo)thermal reactions of (heterocyclicthio)acetic acid with both transition metal ions and lanthanide ions have been investigated in several reports (Zhu *et al.*, 2009; Hao *et al.*, 2008; He *et al.*, 2007), wherein *in situ *C—S cleavage has taken place under these situations. Herein, we report a manganese (II) coordination complex with a newly synthesized (heterocyclicthio)acetic acid, namely 2-(4-(pyridine-3-yl)pyrimidin-2-ylthio)acetic acid.

As shown in Fig. 1, the coordination arrangement around Mn(II) center is similar to our previously reported Cu(II) compound with the ligand of 4-(pyridin-4-yl)pyrimidine-2-sulfonate (Li *et al.*, 2009). The Mn(II) center also adopts an octahedral coordination geometry completed by four water O atoms in equatorial positions and two N atoms in apical positions. In the title complex, the Mn^II^ atom sits on an inversion centre with the asymmetric unit containing half of the complex and one free water molecule. The Mn—O bond lengths vary from 2.189 (2) to 2.192 (2) Å and the Mn—N bond distance is 2.276 (2) Å. Intra- and intermolecular hydrogen bonding interactions, such as O—H···O and O—H···N are observed in the crystal structure (Table 1).

### Experimental

The mixture of Mn(OAc)_2_ (0.1 mmol), 2-(4-(pyridine-3-yl)pyrimidin-2-ylthio)acetic acid (0.2 mmol) and NaOH (0.2 mmol) in 6 ml of H_2_O was stirred for 20 min at room temperature. After filtration, the mother liquid was stood for one week to give the colorless crystals suitable for X-raydiffraction analysis.

### Refinement

C-bound H atoms were positioned geometrically (C—H =0.93 Å) and allowed to ride on their parent atoms, with *U*_iso_(H) = 1.2 *U*_eq_(C). The positions of the water H atoms were found from a difference Fourier map, but placed in idealized positions (O—H = 0.85 Å), and refined as riding with *U*_iso_(H) = 1.2 *U*_eq_(O5).

### Crystal data

**Table:**

[Mn(C_11_H_8_N_3_O_2_S)_2_(H_2_O)_4_]·2H_2_O	*Z* = 1
*M**_r_* = 655.58	*F*(000) = 339
Triclinic, *P*1	*D*_x_ = 1.559 Mg m^−^^3^
Hall symbol: -P 1	Mo *K*α radiation, λ = 0.71073 Å
*a* = 8.459 (3) Å	Cell parameters from 3181 reflections
*b* = 9.240 (3) Å	θ = 2.2–28.1°
*c* = 9.360 (4) Å	µ = 0.69 mm^−^^1^
α = 87.396 (6)°	*T* = 298 K
β = 75.862 (5)°	Block, colourless
γ = 79.872 (5)°	0.14 × 0.12 × 0.10 mm
*V* = 698.4 (4) Å^3^	

### Data collection

**Table:**

Bruker APEXII CCD area-detector diffractometer	3181 independent reflections
Radiation source: fine-focus sealed tube	2443 reflections with *I* > 2σ(*I*)
graphite	*R*_int_ = 0.031
φ and ω scans	θ_max_ = 28.1°, θ_min_ = 2.2°
Absorption correction: multi-scan (*SADABS*; Bruker, 2001)	*h* = −10→10
*T*_min_ = 0.884, *T*_max_ = 0.920	*k* = −6→12
4518 measured reflections	*l* = −11→10

### Refinement

**Table:**

Refinement on *F*^2^	Primary atom site location: structure-invariant direct methods
Least-squares matrix: full	Secondary atom site location: difference Fourier map
*R*[*F*^2^ > 2σ(*F*^2^)] = 0.037	Hydrogen site location: inferred from neighbouring sites
*wR*(*F*^2^) = 0.094	H-atom parameters constrained
*S* = 0.98	*w* = 1/[σ^2^(*F*_o_^2^) + (0.0441*P*)^2^] where *P* = (*F*_o_^2^ + 2*F*_c_^2^)/3
3181 reflections	(Δ/σ)_max_ < 0.001
187 parameters	Δρ_max_ = 0.49 e Å^−^^3^
0 restraints	Δρ_min_ = −0.55 e Å^−^^3^

### Special details

**Table:**

Geometry. All e.s.d.'s (except the e.s.d. in the dihedral angle between two l.s. planes) are estimated using the full covariance matrix. The cell e.s.d.'s are taken into account individually in the estimation of e.s.d.'s in distances, angles and torsion angles; correlations between e.s.d.'s in cell parameters are only used when they are defined by crystal symmetry. An approximate (isotropic) treatment of cell e.s.d.'s is used for estimating e.s.d.'s involving l.s. planes.
Refinement. Refinement of *F*^2^ against ALL reflections. The weighted *R*-factor *wR* and goodness of fit *S* are based on *F*^2^, conventional *R*-factors *R* are based on *F*, with *F* set to zero for negative *F*^2^. The threshold expression of *F*^2^ > σ(*F*^2^) is used only for calculating *R*-factors(gt) *etc*. and is not relevant to the choice of reflections for refinement. *R*-factors based on *F*^2^ are statistically about twice as large as those based on *F*, and *R*- factors based on ALL data will be even larger.

### Fractional atomic coordinates and isotropic or equivalent isotropic displacement parameters (Å^2^)

**Table:**

	*x*	*y*	*z*	*U*_iso_*/*U*_eq_	
Mn1	0.0000	1.0000	0.5000	0.02786 (13)	
S1	0.71353 (7)	0.62719 (6)	0.00440 (6)	0.03937 (16)	
O1	0.12141 (17)	0.98576 (16)	0.26485 (16)	0.0383 (4)	
O2	0.24139 (17)	1.03191 (17)	0.52792 (17)	0.0447 (4)	
N2	0.49123 (19)	0.56249 (17)	0.24128 (18)	0.0291 (4)	
O4	0.44775 (17)	0.95387 (16)	0.25780 (16)	0.0412 (4)	
N1	0.6987 (2)	0.36606 (19)	0.1238 (2)	0.0379 (4)	
C1	0.6227 (2)	0.5074 (2)	0.1385 (2)	0.0310 (4)	
O3	0.71010 (18)	0.85073 (17)	0.23595 (17)	0.0455 (4)	
C5	0.2749 (2)	0.5349 (2)	0.4544 (2)	0.0279 (4)	
N3	0.0768 (2)	0.75451 (18)	0.53326 (19)	0.0335 (4)	
C11	0.5782 (2)	0.8717 (2)	0.1953 (2)	0.0302 (4)	
C4	0.4233 (2)	0.4687 (2)	0.3417 (2)	0.0295 (4)	
C9	0.2097 (2)	0.6826 (2)	0.4396 (2)	0.0323 (5)	
H9A	0.2621	0.7342	0.3595	0.039*	
C10	0.5730 (3)	0.7975 (2)	0.0536 (2)	0.0342 (5)	
H10A	0.5956	0.8662	−0.0272	0.041*	
H10B	0.4614	0.7793	0.0633	0.041*	
C6	0.1962 (3)	0.4588 (2)	0.5742 (2)	0.0354 (5)	
H6A	0.2354	0.3600	0.5882	0.042*	
C8	0.0031 (3)	0.6779 (2)	0.6484 (2)	0.0364 (5)	
H8A	−0.0899	0.7256	0.7149	0.044*	
C7	0.0596 (3)	0.5315 (2)	0.6721 (2)	0.0394 (5)	
H7A	0.0058	0.4824	0.7534	0.047*	
C2	0.6305 (3)	0.2757 (2)	0.2252 (3)	0.0415 (5)	
H2C	0.6786	0.1769	0.2209	0.050*	
C3	0.4920 (3)	0.3206 (2)	0.3369 (3)	0.0402 (5)	
H3A	0.4466	0.2544	0.4060	0.048*	
O5	1.0477 (2)	0.7838 (2)	0.1129 (2)	0.0871 (8)	
H1	0.2712	1.0656	0.5985	0.105*	
H2	0.2259	0.9808	0.2469	0.105*	
H3	0.3309	1.0079	0.4625	0.105*	
H4	0.0999	0.9300	0.2053	0.105*	
H5	1.0980	0.7268	0.0413	0.105*	
H6	0.9449	0.7871	0.1208	0.105*	

### Atomic displacement parameters (Å^2^)

**Table:**

	*U*^11^	*U*^22^	*U*^33^	*U*^12^	*U*^13^	*U*^23^
Mn1	0.0239 (2)	0.0266 (2)	0.0312 (3)	−0.00261 (17)	−0.00359 (17)	−0.00345 (17)
S1	0.0421 (3)	0.0381 (3)	0.0316 (3)	−0.0063 (2)	0.0042 (2)	−0.0080 (2)
O1	0.0354 (8)	0.0416 (9)	0.0347 (8)	−0.0006 (7)	−0.0053 (6)	−0.0082 (6)
O2	0.0289 (8)	0.0619 (11)	0.0447 (10)	−0.0092 (7)	−0.0080 (7)	−0.0149 (8)
N2	0.0291 (9)	0.0264 (9)	0.0310 (9)	−0.0038 (7)	−0.0055 (7)	−0.0033 (7)
O4	0.0305 (8)	0.0442 (9)	0.0447 (9)	−0.0013 (7)	−0.0023 (7)	−0.0141 (7)
N1	0.0360 (10)	0.0308 (10)	0.0452 (11)	0.0008 (8)	−0.0091 (8)	−0.0125 (8)
C1	0.0313 (11)	0.0301 (11)	0.0339 (12)	−0.0060 (8)	−0.0105 (9)	−0.0066 (8)
O3	0.0342 (8)	0.0558 (10)	0.0482 (10)	−0.0005 (7)	−0.0147 (7)	−0.0200 (8)
C5	0.0274 (10)	0.0260 (10)	0.0319 (11)	−0.0056 (8)	−0.0095 (8)	0.0004 (8)
N3	0.0293 (9)	0.0303 (9)	0.0371 (10)	−0.0036 (7)	−0.0019 (7)	−0.0008 (7)
C11	0.0309 (11)	0.0293 (11)	0.0301 (11)	−0.0086 (8)	−0.0039 (8)	−0.0006 (8)
C4	0.0304 (10)	0.0259 (10)	0.0344 (11)	−0.0039 (8)	−0.0125 (9)	−0.0017 (8)
C9	0.0314 (11)	0.0267 (10)	0.0361 (12)	−0.0048 (8)	−0.0035 (9)	0.0027 (8)
C10	0.0415 (12)	0.0325 (11)	0.0283 (11)	−0.0068 (9)	−0.0075 (9)	0.0003 (8)
C6	0.0428 (12)	0.0274 (11)	0.0374 (12)	−0.0076 (9)	−0.0121 (10)	0.0045 (9)
C8	0.0324 (11)	0.0390 (12)	0.0342 (12)	−0.0070 (9)	−0.0003 (9)	−0.0015 (9)
C7	0.0427 (13)	0.0403 (13)	0.0335 (12)	−0.0119 (10)	−0.0036 (10)	0.0084 (9)
C2	0.0462 (13)	0.0246 (11)	0.0512 (14)	0.0041 (10)	−0.0130 (11)	−0.0077 (10)
C3	0.0456 (13)	0.0253 (11)	0.0470 (14)	−0.0031 (9)	−0.0081 (11)	0.0008 (9)
O5	0.0420 (10)	0.1175 (19)	0.0989 (17)	−0.0176 (11)	0.0041 (11)	−0.0692 (14)

### Geometric parameters (Å, °)

**Table:**

Mn1—O1	2.1889 (16)	C5—C9	1.393 (3)
Mn1—O1^i^	2.1889 (16)	C5—C4	1.485 (3)
Mn1—O2^i^	2.1919 (15)	N3—C9	1.335 (2)
Mn1—O2	2.1919 (15)	N3—C8	1.345 (3)
Mn1—N3^i^	2.2761 (18)	C11—C10	1.534 (3)
Mn1—N3	2.2761 (18)	C4—C3	1.388 (3)
S1—C1	1.759 (2)	C9—H9A	0.9300
S1—C10	1.800 (2)	C10—H10A	0.9700
O1—H2	0.8520	C10—H10B	0.9700
O1—H4	0.8477	C6—C7	1.375 (3)
O2—H1	0.8510	C6—H6A	0.9300
O2—H3	0.8511	C8—C7	1.380 (3)
N2—C1	1.320 (2)	C8—H8A	0.9300
N2—C4	1.342 (3)	C7—H7A	0.9300
O4—C11	1.252 (2)	C2—C3	1.382 (3)
N1—C2	1.327 (3)	C2—H2C	0.9300
N1—C1	1.347 (2)	C3—H3A	0.9300
O3—C11	1.246 (2)	O5—H5	0.8500
C5—C6	1.386 (3)	O5—H6	0.8501
			
O1—Mn1—O1^i^	180.000 (1)	C8—N3—Mn1	123.89 (14)
O1—Mn1—O2^i^	95.09 (6)	O3—C11—O4	125.43 (18)
O1^i^—Mn1—O2^i^	84.91 (6)	O3—C11—C10	118.76 (18)
O1—Mn1—O2	84.91 (6)	O4—C11—C10	115.76 (18)
O1^i^—Mn1—O2	95.09 (6)	N2—C4—C3	120.38 (18)
O2^i^—Mn1—O2	180.00 (8)	N2—C4—C5	115.60 (16)
O1—Mn1—N3^i^	87.81 (6)	C3—C4—C5	124.02 (18)
O1^i^—Mn1—N3^i^	92.19 (6)	N3—C9—C5	124.01 (18)
O2^i^—Mn1—N3^i^	88.48 (6)	N3—C9—H9A	118.0
O2—Mn1—N3^i^	91.52 (6)	C5—C9—H9A	118.0
O1—Mn1—N3	92.19 (6)	C11—C10—S1	116.57 (14)
O1^i^—Mn1—N3	87.81 (6)	C11—C10—H10A	108.1
O2^i^—Mn1—N3	91.52 (6)	S1—C10—H10A	108.1
O2—Mn1—N3	88.48 (6)	C11—C10—H10B	108.1
N3^i^—Mn1—N3	180.000 (1)	S1—C10—H10B	108.1
C1—S1—C10	101.21 (10)	H10A—C10—H10B	107.3
Mn1—O1—H2	113.5	C7—C6—C5	119.14 (19)
Mn1—O1—H4	123.8	C7—C6—H6A	120.4
H2—O1—H4	108.6	C5—C6—H6A	120.4
Mn1—O2—H1	132.6	N3—C8—C7	122.84 (19)
Mn1—O2—H3	122.8	N3—C8—H8A	118.6
H1—O2—H3	104.6	C7—C8—H8A	118.6
C1—N2—C4	117.31 (17)	C6—C7—C8	119.36 (19)
C2—N1—C1	114.58 (18)	C6—C7—H7A	120.3
N2—C1—N1	127.05 (19)	C8—C7—H7A	120.3
N2—C1—S1	118.41 (15)	N1—C2—C3	123.44 (19)
N1—C1—S1	114.55 (15)	N1—C2—H2C	118.3
C6—C5—C9	117.58 (18)	C3—C2—H2C	118.3
C6—C5—C4	124.11 (18)	C2—C3—C4	117.2 (2)
C9—C5—C4	118.31 (17)	C2—C3—H3A	121.4
C9—N3—C8	117.06 (18)	C4—C3—H3A	121.4
C9—N3—Mn1	118.88 (13)	H5—O5—H6	106.5
			
C4—N2—C1—N1	−0.9 (3)	C9—C5—C4—C3	−174.50 (19)
C4—N2—C1—S1	179.48 (14)	C8—N3—C9—C5	0.1 (3)
C2—N1—C1—N2	0.1 (3)	Mn1—N3—C9—C5	175.57 (15)
C2—N1—C1—S1	179.79 (15)	C6—C5—C9—N3	−0.1 (3)
C10—S1—C1—N2	−3.72 (17)	C4—C5—C9—N3	179.86 (18)
C10—S1—C1—N1	176.59 (14)	O3—C11—C10—S1	28.1 (3)
O1—Mn1—N3—C9	24.51 (15)	O4—C11—C10—S1	−154.45 (16)
O1^i^—Mn1—N3—C9	−155.49 (15)	C1—S1—C10—C11	71.70 (17)
O2^i^—Mn1—N3—C9	119.67 (15)	C9—C5—C6—C7	−0.2 (3)
O2—Mn1—N3—C9	−60.33 (15)	C4—C5—C6—C7	179.86 (19)
O1—Mn1—N3—C8	−160.35 (16)	C9—N3—C8—C7	0.2 (3)
O1^i^—Mn1—N3—C8	19.65 (16)	Mn1—N3—C8—C7	−175.02 (15)
O2^i^—Mn1—N3—C8	−65.20 (17)	C5—C6—C7—C8	0.5 (3)
O2—Mn1—N3—C8	114.80 (17)	N3—C8—C7—C6	−0.5 (3)
C1—N2—C4—C3	0.9 (3)	C1—N1—C2—C3	0.6 (3)
C1—N2—C4—C5	−179.45 (16)	N1—C2—C3—C4	−0.5 (3)
C6—C5—C4—N2	−174.15 (19)	N2—C4—C3—C2	−0.3 (3)
C9—C5—C4—N2	5.9 (3)	C5—C4—C3—C2	−179.9 (2)
C6—C5—C4—C3	5.5 (3)		

Symmetry codes: (i) −*x*, −*y*+2, −*z*+1.

### Hydrogen-bond geometry (Å, °)

**Table:**

*D*—H···*A*	*D*—H	H···*A*	*D*···*A*	*D*—H···*A*
O2—H1···O3^ii^	0.85	1.82	2.655 (2)	168
O1—H2···O4	0.85	1.88	2.709 (2)	165
O2—H3···O4	0.85	1.97	2.743 (2)	150
O1—H4···O5^iii^	0.85	1.81	2.642 (3)	167
O5—H5···N1^iv^	0.85	2.09	2.888 (3)	155
O5—H6···O3	0.85	2.01	2.775 (3)	149

Symmetry codes: (ii) −*x*+1, −*y*+2, −*z*+1; (iii) *x*−1, *y*, *z*; (iv) −*x*+2, −*y*+1, −*z*.
